# Methylprednisolone Modulates the Tfr/Tfh ratio in EAE-Induced Neuroinflammation through the PI3K/AKT/FoxO1 and PI3K/AKT/mTOR Signalling Pathways

**DOI:** 10.1007/s10753-024-02099-y

**Published:** 2024-07-09

**Authors:** Nan Wu, Yun Zhao, Minjun Xiao, Hui Liu, Hongliang Chen, Bin Liu, Xuezhen Wang, Xueli Fan

**Affiliations:** 1 Department of Neurology, Binzhou Medical University Hospital, Binzhou, China; 2 Institute for Metabolic & Neuropsychiatric Disorders, Binzhou Medical University Hospital, Binzhou, China

**Keywords:** multiple sclerosis (MS), experimental autoimmune encephalomyelitis (EAE), T follicular helper (Tfh) cells, T follicular regulatory (Tfr) cells, PI3K/AKT/FoxO1 signalling pathway, PI3K/AKT/mTOR signalling pathway

## Abstract

Methylprednisolone (MP) is a potent glucocorticoid that can effectively inhibit immune system inflammation and brain tissue damage in Multiple sclerosis (MS) patients. T follicular helper (Tfh) cells are a subpopulation of activated CD4 + T cells, while T follicular regulatory (Tfr) cells, a novel subset of Treg cells, possess specialized abilities to suppress the Tfh-GC response and inhibit antibody production. Dysregulation of either Tfh or Tfr cells has been implicated in the pathogenesis of MS. However, the molecular mechanism underlying the anti-inflammatory effects of MP therapy on experimental autoimmune encephalomyelitis (EAE), a representative model for MS, remains unclear. This study aimed to investigate the effects of MP treatment on EAE and elucidate the possible underlying molecular mechanisms involed. We evaluated the effects of MP on disease progression, CNS inflammatory cell infiltration and myelination, microglia and astrocyte activation, as well as Tfr/Tfh ratio and related molecules/inflammatory factors in EAE mice. Additionally, Western blotting was used to assess the expression of proteins associated with the PI3K/AKT pathway. Our findings demonstrated that MP treatment ameliorated clinical symptoms, inflammatory cell infiltration, and myelination. Furthermore, it reduced microglial and astrocytic activation. MP may increase the number of Tfr cells and the levels of cytokine TGF-β1, while reducing the number of Tfh cells and the levels of cytokine IL-21, as well as regulate the imbalanced Tfr/Tfh ratio in EAE mice. The PI3K/AKT/FoxO1 and PI3K/AKT/mTOR pathways were found to be involved in EAE development. However, MP treatment inhibited their activation. MP reduced neuroinflammation in EAE by regulating the balance between Tfr/Tfh cells via inhibition of the PI3K/AKT/FoxO1 and PI3K/AKT/mTOR signalling pathways.

**Supplementary Information:**

The online version contains supplementary material available at 10.1007/s10753-024-02099-y.

## INTRODUCTION

Multiple sclerosis (MS) is one of the most prevalent chronic autoimmune diseases involving the central nervous system (CNS), which leads to focal lesions in the white and grey matter, as well as diffuse neurodegeneration throughout the brain [1]. Experimental autoimmune encephalomyelitis (EAE), induced by myelin oligodendrocyte glycoprotein (MOG), is the most common animal model used for the study of MS [2]. The pathological manifestations of EAE include inflammatory cell infiltration and demyelination in the white matter of the brain and spinal cord, which are very similar to those of MS [3]. Additionally, MS is the most common nontraumatic disabling disease affecting more than 2.5 million people worldwide especially young adults between 20 and 40 years of age. MS affects women nearly two to three times more often than men [4]. It is characterized by completely or partially reversible episodes of neurologic symptoms that generally last days or weeks [5]. The typical clinical presentations of MS include visual loss, limb weakness or sensory disturbances, double vision, ataxia, spasticity, and cognitive deficits, depending on the location of the lesions [6]. Currently, MS is incurable because no medication can fully prevent or reverse the progressive neurological deficits [5].

To date, the underlying cause and mechanisms of MS remain unclear [7]. Recently, MS has been regarded as a heterogeneous and multifactorial disease caused by multiple genetic and environmental risk factors, including alleles of the genes encoding the major histocompatibility complex, interleukin-2 receptor, interleukin-7 receptor), and smoking, vitamin D deficiency, Epstein-Barr virus infection, diet and obesity [8]. Except for the particular triggering cause, a dysregulated immune response is a key player in the pathogenesis of MS [9]. Different subsets of CD4 + T cells orchestrate an inflammatory response to initiate the pathogenesis of MS [10, 11]. In addition to T cells, B cells and innate immunity also participate in the onset of MS. B cells produce a specific IgG oligoclonal band present in the cerebrospinal fluid, which is one of the diagnostic criteria for MS [12]. T follicular helper (Tfh) cells, a helper T (Th) cell subset, are essential for the differentiation of B cells into memory B cells in germinal centers (GCs), and can promote the production of autoantibodies by antibody-producing plasma cells [13]. Besides, Tfh cells not only express the chemokine receptor CXCR5, inducible T-cell costimulator (ICOS), and programmed death-1 (PD-1) but also secrete cytokines, including IL-6, and IL-21 [14, 15].

Recently discovered follicular regulatory (Tfr) cells are considered negative regulators of GC responses due to their secretion of anti-inflammatory cytokines, including IL-10 and TGF-β1 [16]. These dual-functioning subpopulations possess characteristics from both Tfh and regulatory T (Treg) lineages. On one hand, they express typical molecules associated with the Tfh lineage, such as CXCR5, ICOS, PD-1, and Bcl-6. On the other hand, Tfr cells uniquely express Foxp3, the master regulator of Tregs, as well as other Treg-related molecules, such as cytotoxic T-lymphocyte-associated protein 4 (CTLA-4) and CD25 [17, 18]. Furthermore, there is a dynamic equilibrium between Tfr and Tfh cells. Several studies have demonstrated that an imbalance in the ratio of Tfr/Tfh cells may result in the excessive production of self-reactive autoantibodies, thereby promoting the occurrence of autoimmune disorders such as systemic lupus erythematosus(SLE) and rheumatoid arthritis (RA) and myasthenia gravis (MG) [19–21]. Hence, regulating this ratio may confer therapeutic benefits for autoimmune diseases.

Recent findings suggest that the secretion of reactive oxygen species, chemokines, and cytokines by infiltrating macrophages and activated microglia within the central nervous system plays a crucial role in the pathogenesis of MS and EAE [22]. In MS pathology, microglia undergo activation leading to polarization towards, the M1 or M2 phenotype. Notably, an increase in the M1 phenotype population contributes to myelin damage along with axonal and neuronal impairment. Conversely, an increase in the M2 phenotype population aids disease alleviation [23]. Additionally, astrocytic neuroinflammation is closely associated with MS pathogenesis. Hyperactivated astrocytes induce inflammatory mediator generation. Bidirectional crosstalk between microglia and astrocytes occurs largely through inflammatory mediators. IL-1β is secreted by M1-type microglia and can activate astrocytes that are harmful in MS [24, 25].

The phosphatidylinositol-3-kinase (PI3K) pathway plays a core role in cell growth, and differentiation and the cell cycle, and AKT serves as a direct downstream effector molecule for the PI3K signalling cascade. Moreover, PI3K/AKT pathway plays an essential role in the process and release of proinflammatory factors [26]. Forkhead box protein 1 (FoxO1), which is encoded by the FoxO1 gene and belongs to the Forkhead box (Fox) family, is regulated primarily via the phosphorylation of numerous residues.FoxO1 and mTOR are key downstream targets of the PI3K/AKT signalling pathway [27]. FoxO1 has been found to control various downstream genes, such as those involved in inflammation, those involved in cell adhesion, regulators of B cells, and modulators of T-regulatory cells [28]. Evidence suggests that targeting the mTOR pathway holds promise as a potential therapeutic strategy for rheumatoid arthritis (RA) and other autoimmune diseases, offering the opportunity to restore Tfr cell functionality and quantity and thereby addressing underlying pathogenic mechanisms. Indeed, baicalin exerts its effects by rebalancing the ratio betweenTfr and Tfh cells through suppression of the mTOR signalling pathway, resulting in amelioration of lupus nephritis symptoms [29]. Methylprednisolone (MP) is a potent glucocorticosteroid which effectively inhibits inflammation and allergic reactions in the immune system [30]. MP can inhibit T-cell activation, reduce inflammatory cytokines levels, and decrease immune cell migration into the CNS to alleviate brain tissue injury in MS patients [31]. Despite these advances, whether MP can inhibit inflammation by regulating the Tfr/Tfh cell ratio balance in the context of EAE remains unclear. Besides, whether the protective effect of MP is correlated with the PI3K/AKT/FoxO1 and PI3K/AKT/mTOR signalling pathways remains largely undefined.

In this study, we aimed to provide insights into the underlying mechanisms of the pathogenesis of EAE.

## MATERIALS AND METHODS

### Animals

Female C57BL/6 mice of specific pathogen-free (SPF) level, aged 6–8 weeks and weighing 19–21 g, were purchased from Jinan Pengyue Laboratory Animal Breeding Co., Ltd. (Ji nan, China). Each animal was bred under SPF conditions with a humidity of 60%-80% and an environmental temperature of 22 to 26 ℃ and had ad libitum access to food and water. All animals used in the study were treated in accordance with the guidelines set forth by the Institutional Animal Care and Use Committee of Binzhou Medical University Hospital.

### Establishment of the EAE model and clinical evaluation

Five milligrams of MOG35-55 polypeptide (GenScript, New Jersey, USA) was added to 2.5 ml of PBS at a final concentration of 2 mg/ml. Then, 17.5mg of heat-killed Mycobacterium tuberculosis ( Becton, Dickinson and Company Sparks, USA) was dissolved in 2.5 ml of complete Freund's adjuvant (CFA, Sigma-Aldrich, Missouri, USA), for a final concentration of 5 mg/ml. The two solutions were thoroughly mixed at a ratio of 1:1. Then, the antigen was fully emulsified on ice to form a water-in-oil emulsion. Each mice was immunized subcutaneously on the back with 0.2 ml of polypeptide. On Day 0 and Day 2 after immunization, the mice received intraperitoneal injections of pertussis toxin (PTX, Merck Millipore Corp, USA) at a dose of 300 ng per injection.

Clinical signs were evaluated daily using an established scoring system ranging from no clinical signs to tetraplegia or moribund by EAE: 0, no clinical signs; 0.5, limp distal tail; 1, paralyzed or staggering tail; 1.5, limp tail and hind limb weakness; 2, mild paresis of both hind limbs or severe paralysis of one hind limb; 3, complete paralysis of both hind limbs; 4, paralyzed limbs and forelimbs; and 5, tetraplegia or moribund by EAE. The intermediate clinical signs in between the major clinical signs were scored as 0.5 points.

### Treatment of EAE mice

All C57BL/6 mice were randomized assigned into 3 groups: (A) the control group, normal mice; (B) EAE group, saline solution-treated EAE mice; and (C) EAE + MP group, MP-treated EAE mice. Treatment with MP (Sinopharm Ronshyn Pharmaceutical Co., Ltd.) was administered once every day from the 9th day (early onset of EAE) to the end of the experiment. The dose was gradually reduced over 9 days, starting at 100 mg/kg for 3 days, followed by 50 mg/kg for another 3 days, and finally 25 mg/kg for the remaining 3 days. Intraperitoneal injections were stopped on the 18th day of the experiment. Control mice received equal volumes of saline solution.

### Histopathological analysis

Histopathological examination was performed on Day19 post-immunization). The spinal cord tissues were meticulously dissected from the mice, fixed in 4% paraformaldehyde, dehydrated, paraffin-embedded, and sectioned into 6 μm-thick sections. Subsequently, spinal cord sections were stained not only with hematoxylin and eosin (H&E) (Solebo Biotech, Beijing, China) to evaluate inflammation but also with luxol fast blue (LFB) (Solebo Biotech, Beijing, China) to assess demyelination. These sections were then scanned and imaged with a microscope (Olympus, Japan), and the inflammatory cell infiltration and demyelination of the spinal cord in the mice were analysed in a blinded fashion. The inflammation scale was determined based on the presence of inflammatory cells: 0 indicated no inflammatory cells, 1 denoted a few scattered inflammatory cells, 2 represented organized infiltration around blood vessels, and 3 signified extensive perivascular cuffing with extension into the surrounding tissue. Demyelination assessment in the spinal cord followed this scale: 0 indicated the absence of demyelination, 1 represented rare focal areas, 2 denoted multiple demyelinated regions, and 3 signified large areas exhibiting demyelination [32, 33].

### Flow cytometry

For cell phenotype analysis, anti-CD4-FITC, anti-CXCR5-percp-cy5.5, anti-Foxp3-PE, anti-ICOS-APC, anti-PD-1-APC antibodies as well as the corresponding IgG isotypes were obtained from BioLegend (San Diego, CA, USA).

For cell surface staining, single cells from the spleens of EAE mice treated with saline solution or MP were incubated with fluorescent monoclonal antibodies at 4 ℃ for 30 min at the optimal dilutions. After cell surface staining, a Foxp3 Staining Buffer Set (Invitrogen) for intracellular staining of cells was used to fix and permeabilize the cells at room temperature for 1h. After the incubation period, the cells were washed twice with PBS supplemented with 2% foetal bovine serum. We determined the panels according to isotype control (Supplementary Fig. 1) and simple staining (Supplementary Fig. 2) of ICOS, PD-1, and Foxp3. Flow cytometry analysis was conducted using a FACS Calibur flow cytometer (Beckman Coulter). The obtained data were analysed utilizing FlowJo 10.0 software.

### Immunofluorescence staining

Mice were anaesthetized and sequentially transcardially perfused with cold PBS and 4% paraformaldehyde (PFA) after which the spinal cord and spleen were removed. The tissues were fixed in 4% paraformaldehyde for 24 h, and then dehydrated in 30% sucrose for 2 days.The sections were obtained using a freezing microtome (Leica, CM1950). For fluorescence staining, the sections were permeabilized with PBS containing 0.4% Triton X-100 (BOSTER, China). Subsequently, the spinal cord/spleen sections were blocked with 5% bovine serum albumin (BSA, BOSTER; China) at room temperature for one hour. Next, the sections were incubated overnight at 4 °C with primary antibodies against GFAP (Cell Signaling Technology), Iba-1 (Cell Signaling Technology), CD4 (Santa Cruz), CXCR5 (BioLegend), and Foxp3 (ABclonal). The sections were then incubated with Alexa Fluor 488-conjugated secondary antibodies (Cell Signaling Technology), Goat anti-Rabbit; Alexa Fluor 546, goat anti-rat (Cell Signaling Technology); Alexa Fluor 647, goat anti-mouse (Santa Cruz) at room temperature for one hour. Next, the nuclei were stained with DAPI before being imaged. Images were captured with an Olympus FV10 confocal microscope (Olympus, Japan).

### Western blot analysis

Spinal cord tissues were dissected from each group. For the EAE group, the spinal cord samples were carefully dissected on Day 9 post-immunization (preimmune to EAE, Pre-EAE group) and Day19 postimmunization (peak of EAE, P-EAE group) and frozen immediately in liquid nitrogen. The spinal cord was subjected to protein extraction using RIPA lysis buffer supplemented with phosphatase and protease inhibitors. The concentration of proteins was determined by employing the BCA assay (BOSTER, China). Protein samples were added to each well for electrophoresis separation via SDS-PAGE (80 V, 0.5 h and then 120 V, 1.5 h). The separated protein bands were transferred to 0.45 μm PVDF membranes (Servicebio, China). The membranes were blocked in a solution containing TBST with 5% skim milk for one hour and then incubated respectively with anti-PI3K (Cell Signaling Technology, 34,050), anti-phospho-AKT (Affinity, AF0016), anti-AKT (Affinity, AF6261), anti-phospho-FoxO1 (Cell Signaling Technology, 9464), anti-FoxO1(Affinity, AF3417), anti-phospho-mTOR (Cell Signaling Technology, 5536), anti-mTOR (Cell Signaling Technology, 2983), CXCR5 (ABclonal, A8950), Foxp3 (Affinity, AF6544), ICOS (ABclonal, A1811), PD-1 (Cell Signaling Technology, 84,651), TGF-β1 (Proteintech, 21,898–1-AP), IL-21 (Affinity, DF4818), GFAP (Cell Signaling Technology, 3670), Iba-1 (Cell Signaling Technology, 17,198), Arg-1 (Proteintech,16,001–1-AP), iNOS (Proteintech, 18985–1-AP) and GAPDH (BOSTER, BM3874) antibodies overnight at 4 ℃. The membranes were incubated with an anti-rabbit antibody conjugated with HRP (Boster, BA1054) for one hour. Finally, ECL reagent (Boster, China) was added, and the protein bands were detected by a chemiluminescence imaging system (LI-COR Biosciences). The band density was quantified using image J.

### Statistical analysis

The data for each group are presented as the mean ± standard error of the mean(SEM). Statistical analyses were conducted using GraphPad Prism, V8.0 (GraphPad Software Inc., San Diego, USA). To calculate differences between two groups, the statistical Student’s t test was used for statistical analysis, while for comparisons among multiple groups, one-way analysis of variance (ANOVA) was used, followed by Bonferroni multiple comparison tests. In the MOG-EAE experiments, the statistical significance of differences in neurological scores was determined using two-way ANOVA. Significant differences between or among groups were assessed accordingly: * *P* < 0.05, ** *P* < 0.01, and *** *P* < 0.001.

## RESULTS

### MP reduced the severity of EAE

**Fig. 1:**
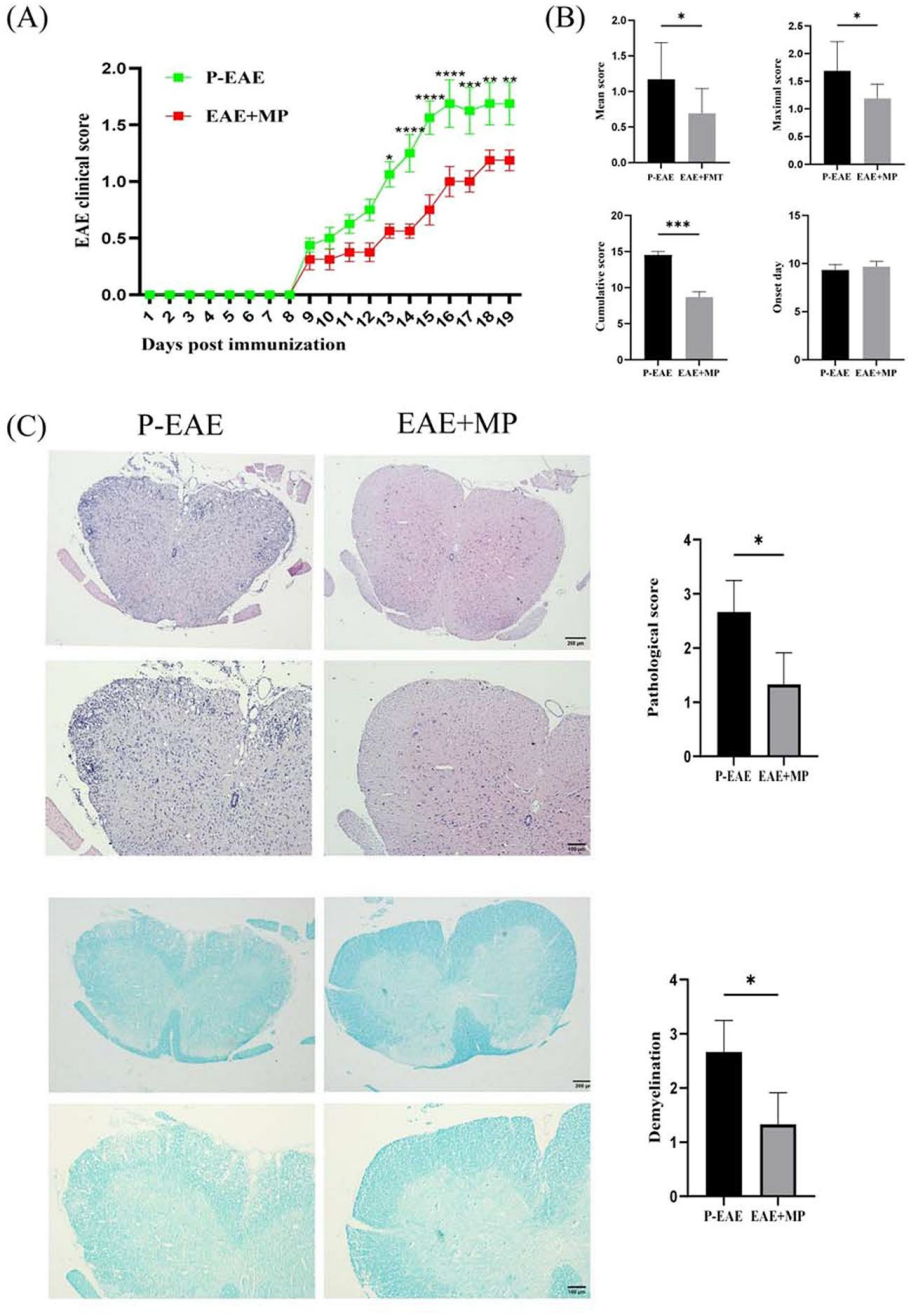
MP reduced the clinical and histopathological scores of EAE. **A** Daily fluctuations in clinical scores between the two cohorts throughout the course of the disease. (mean ± SEM; n = 8 each; * *P* < 0.05, ** *P* < 0.01, and *** *P* < 0.001). **B** Analysis of maximal scores, cumulative scores, and mean scores as well as onset day for both the P-EAE group and MP group. (*n* = 8 each; * *P* < 0.05, ** *P* < 0.01, and *** *P* < 0.001). **C** Inflammatory infiltration observed through H&E and demyelination detected by LFB staining with corresponding inflammation scores. (*n* = 3 each; * *P* < 0.05, ** *P* < 0.01, and *** *P* < 0.001).

To evaluate the therapeutic efficacy of MP on EAE, we examined the clinical score of mice from Day 0 to Day 19 after immunization. Neurological deficits became apparent around the 9th day after immunization and progressively worsened, eaching a peak around the 16th day after immunization. The clinical scores of the EAE + MP group were significantly lower than those of the EAE group (*P* < 0.05, Fig. 1). The EAE + FMT group significantly lower cumulative, maximum, and mean EAE scores than did the EAE group. However, there was no significant difference between the onset days of the EAE and EAE + MP groups (Fig. 1).

### MP attenuated inflammatory cell infiltration and demyelination in the spinal cords of EAE mice

To evaluate the extent of inflammatory cell infiltration and remyelination in the lumbar spinal cord of EAE mice, H&E and LFB staining were performed. We observed decreased perivascular and parenchymal infiltrated mononuclear cells within white matter as well as reduced perivascular cuffs in the EAE + MP group compared to the P-EAE group (*P* < 0.05, Fig. 1C). Additionally, LFB staining revealed less extensive demyelination in the spinal cords of mice treated with MP than in those of untreated mice (*P* < 0.05, Fig. 1C). These findings indicate that MP effectively alleviates inflammatory cell infiltration and demyelination in a murine model of EAE.

### MP ameliorated EAE by suppressing microglial and astrocytic activation

**Fig. 2:**
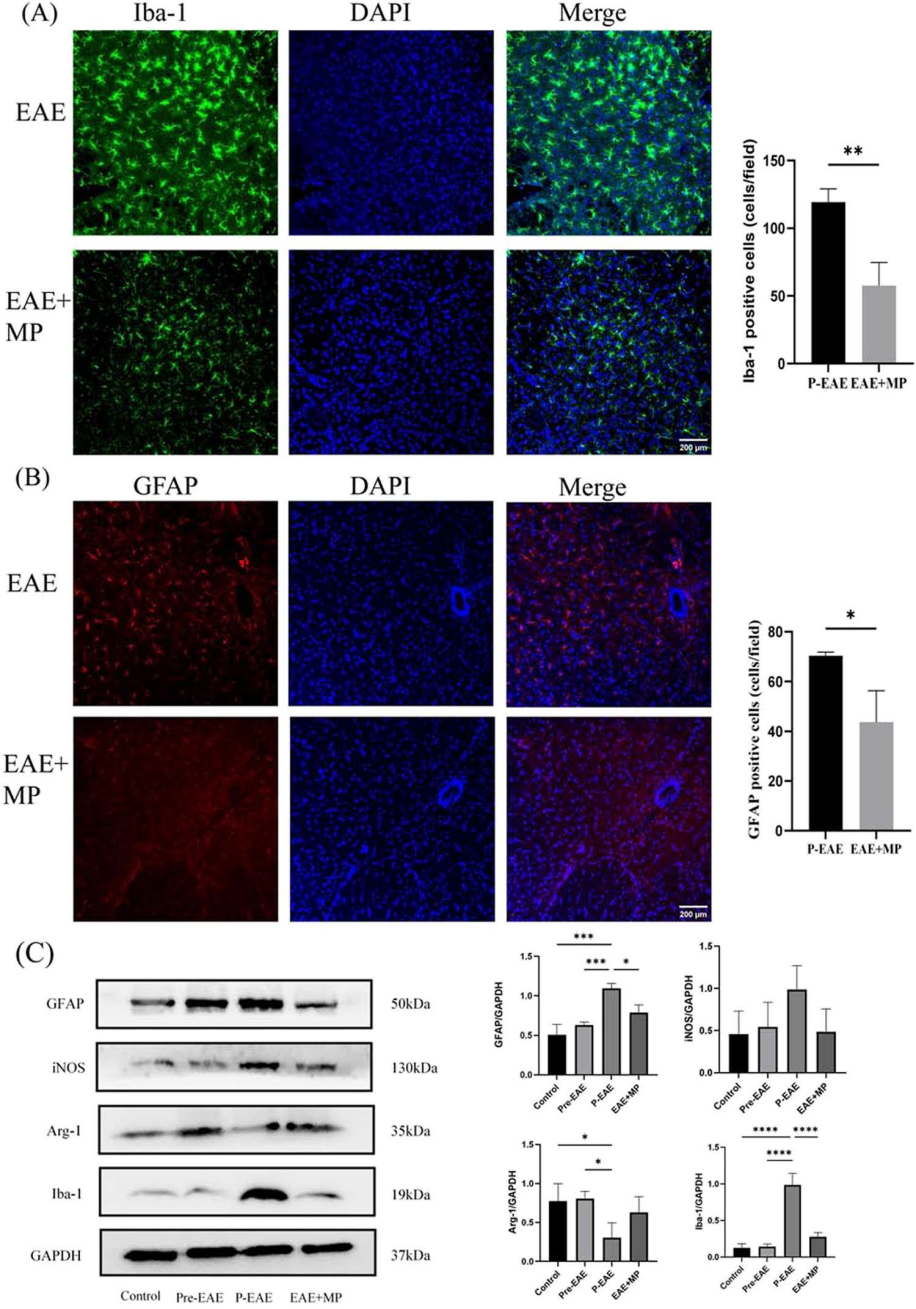
MP inhibited microglial and astrocytic activation in EAE mice. **A**-B The expression of the anti-GFAP and anti-Iba-1 protein in the lumbar spinal cords of the P-EAE, and EAE + MP groups, was detected by immunofluorescence staining. *n* = 3 per group. The data are presented as the mean ± SEM. *P < 0.05, **P < 0.01 **C** The protein expression levels of GFAP, Iba-1, iNOS, and Arg-1 were investigated in lysates from lumbar spinal cords via Western blot analysis. *n* = 3 per group. The data are presented as the mean ± SEM. * *P* < 0.05, *** *P* < 0.001.

Microglial and astrocytic activation promotes the production of proinflammatory cytokines and inflammatory cell infiltration in the CNS of EAE animals [34]. We evaluated the expression of glial fibrillary acidic protein (GFAP), a marker for astrocytes, and ionized calcium binding adaptor protein (Iba-1), a marker of microglia, through immunofluorescence analysis of lumbar spinal cord sections. The mice in the EAE + MP group exhibited significantly fewer Iba-1-positive microglia than did those in the P-EAE group (*P* < 0.05, Fig. 2A). Moreover, there was a decrease in GFAP-positive astrocytes observed in the spinal cord in the MP-treated EAE group compared with the P-EAE group (*P* < 0.05, Fig. 2B). Western blot analysis revealed significant upregulation of GFAP expression in the P-EAE group compared to both the control and Pre-EAE groups. However, MP treatment abrogated this increase in P-EAE group (*P* < 0.05, Fig. 2C). Additionally, iNOS protein levels showed a trend consistent with that of Iba-1 among the different groups, indicating an anti-inflammatory effect, but the difference was not statistically significant (Fig. 2C). Furthermore, Arg-1 protein levels decreased significantly in the P-EAE group as compared to those in the control and Pre-EAE groups; however, MP treatment abrogated this decrease without statistical significance. These findings suggest that MP can ameliorate EAE by suppressing microglial and astrocytic activation in vivo while reducing inflammatory responses and promoting anti-inflammatory responses by inhibiting M1 microglial activity as well as promoting polarization towards the M2 phenotype.

### MP decreased Tfh cell abundance, increased Tfr cell abundance, and restores the balance of the Tfr/Tfh cell ratio in EAE mice

**Fig. 3:**
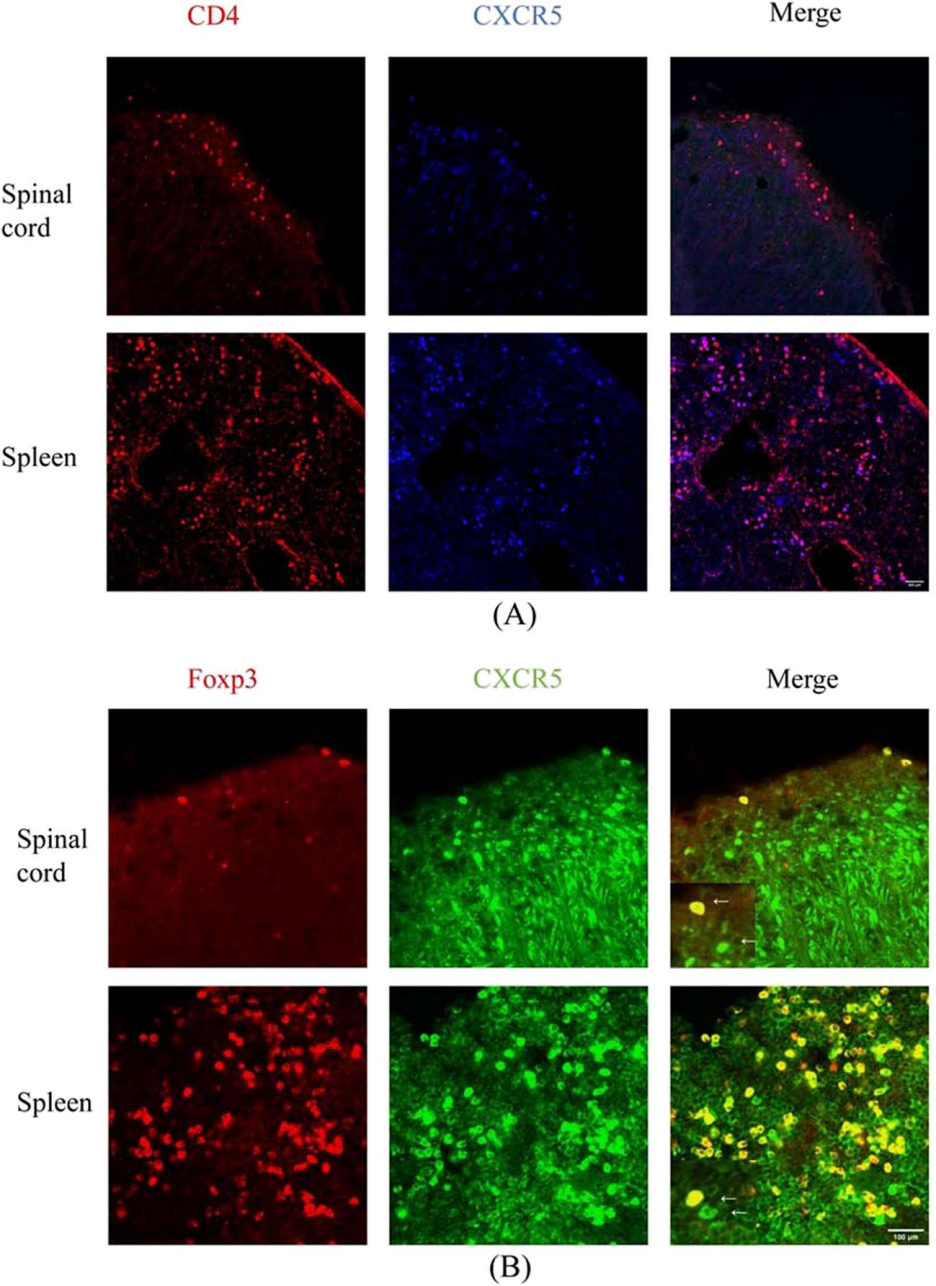
Confocal photomicrographs of Tfh (**A**) and Tfr (**B**) cell localization in the spleens and spinal cord of the P-EAE group. Sections are representative of three mice analyzed.

**Fig. 4:**
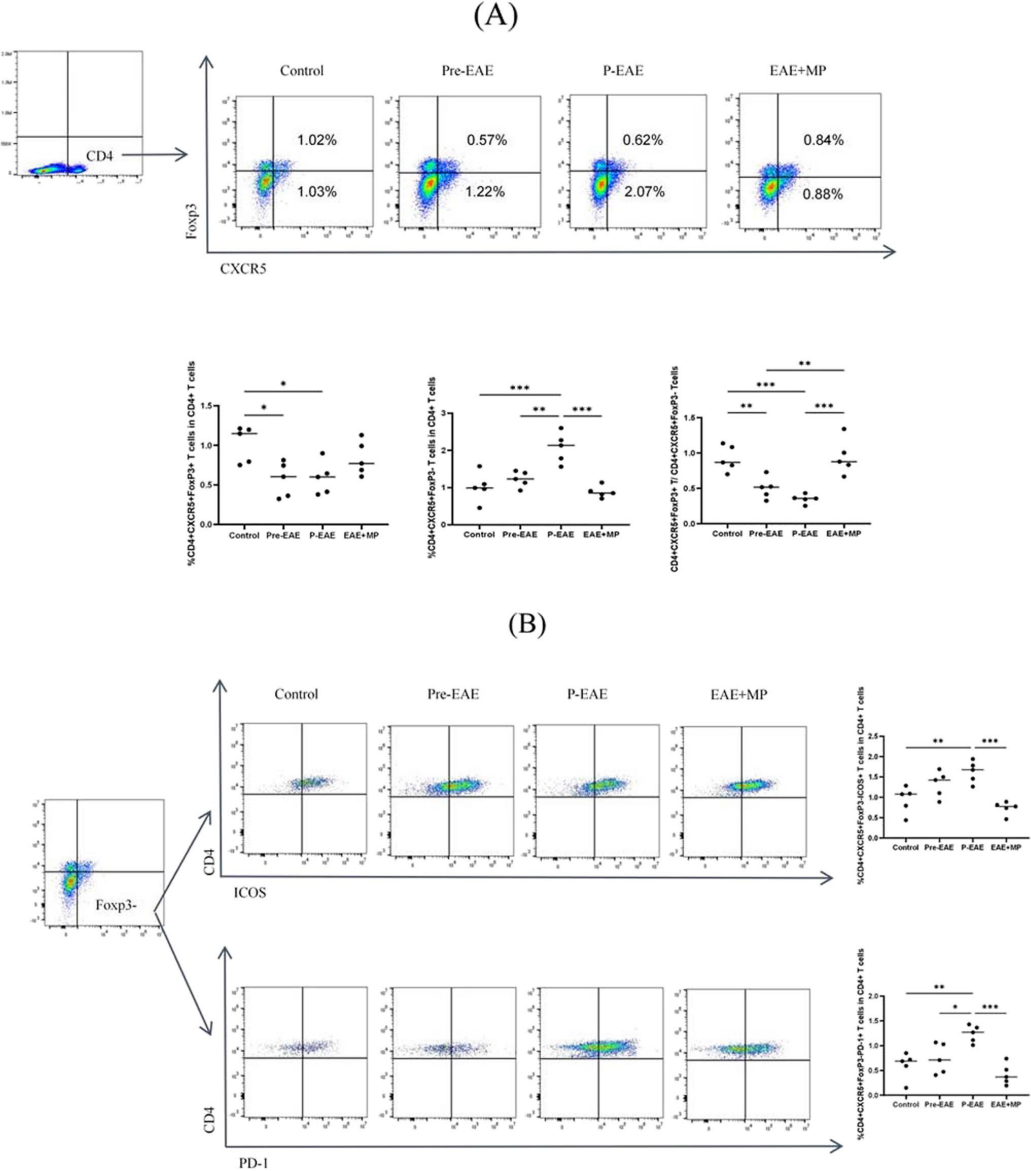
Flow cytometry analysis of different subsets of splenic Tfr and Tfh cells in the control, Pre-EAE, P-EAE, and EAE + MP groups. **A** The percentages of CD4 + CXCR5 + Foxp3 + Tfr and CD4 + CXCR5 + Foxp3-Tfh cells in the spleens of EAE mice in these four groups. **B** The percentages of CD4 + CXCR5 + Foxp3-ICOS + and CD4 + CXCR5 + Foxp3-PD-1 + Tfh cells in the spleens of EAE mice in these four groups. *n* = 5 per group. The data are presented as the mean ± SEM. * *P* < 0.05, ** *P* < 0.01*** *P* < 0.001.

Studies have shown that CD4 + CXCR5 + Tfh-like cells are located at the T-B border [35]. The Tfr cells were positive for Foxp3 expression, while the Tfh cells were negative [36, 37]. We determined the location of CD4 + CXCR5 + Tfh cells, CXCR5 + Foxp3 + Tfr cells and CXCR5 + Foxp3-Tfh cells in the spleen and spinal cord (Fig. 3) using immunofluorescence staining. CD4 + CXCR5 + Tfh cells were present within the inflammatory area in the spinal cords and GCs in the spleens of EAE mice. CXCR5 + Foxp3 + Tfr and CXCR5 + Foxp3-Tfh cells also existed in areas where CD4 + CXCR5 + Tfh cells were present. To further investigate the systemic immunoregulatory effects of MP, we performed flow cytometry analysis on individual spleen cells. Tfh cells were defined as CD4 + CXCR5 + Foxp3-, and their percentage increased from an average of 1.22% in Pre-EAE mice to 2.07% in P-EAE mice. However, MP treatment significantly reduced the percentage of Tfh cells to anaverage of 0.88% (Fig. 4B), indicating its inhibitory effect on Tfh cell abundance in EAE mice. In addition, the percentage of CD4 + CXCR5 + Foxp3 + Tfr cells was lower in both the Pre-EAE and P-EAE groups than in the control group (*P* < 0.05, Fig. 4B). Nevertheless, MP treatment partially increased the percentage of Tfr cells. The ratio of Tfr/Tfh cells was significantly lower in both the Pre-EAE and P-EAE groups than in the control group but was reversed after treatment with MP (*P* < 0.05, Fig. 4B). We observed an increase in the percentage of the Tfh cell subtype, identified as CD4 + CXCR5 + Foxp3-ICOS + , which increased in Pre-EAE, reached a maximum in P-EAE, and decreased after MP treatment (*P* < 0.05, Fig. 4B). The proportion of CD4 + CXCR5 + Foxp3-PD-1 Tfh cells in the P-EAE group was significantly higher than that in the control group and the Pre-EAE group. Compared to the P-EAE group, MP treatment significantly reduced the percentage of Tfh cells (*P* < 0.05, Fig. 4B). Our findings suggest that MP treatment can correct the imbalance between Tfr and Tfh cells by decreasing the abundance of Tfh cells and increasing the abundance of Tfr cells, thereby exerting its systemic immunoregulatory effect on EAE mice.

### MP treatment resulted in the downregulation of Tfh-related molecules and proinflammatory factors while promoting the expression of Tfr-related molecules and anti-inflammatory factors

**Fig. 5:**
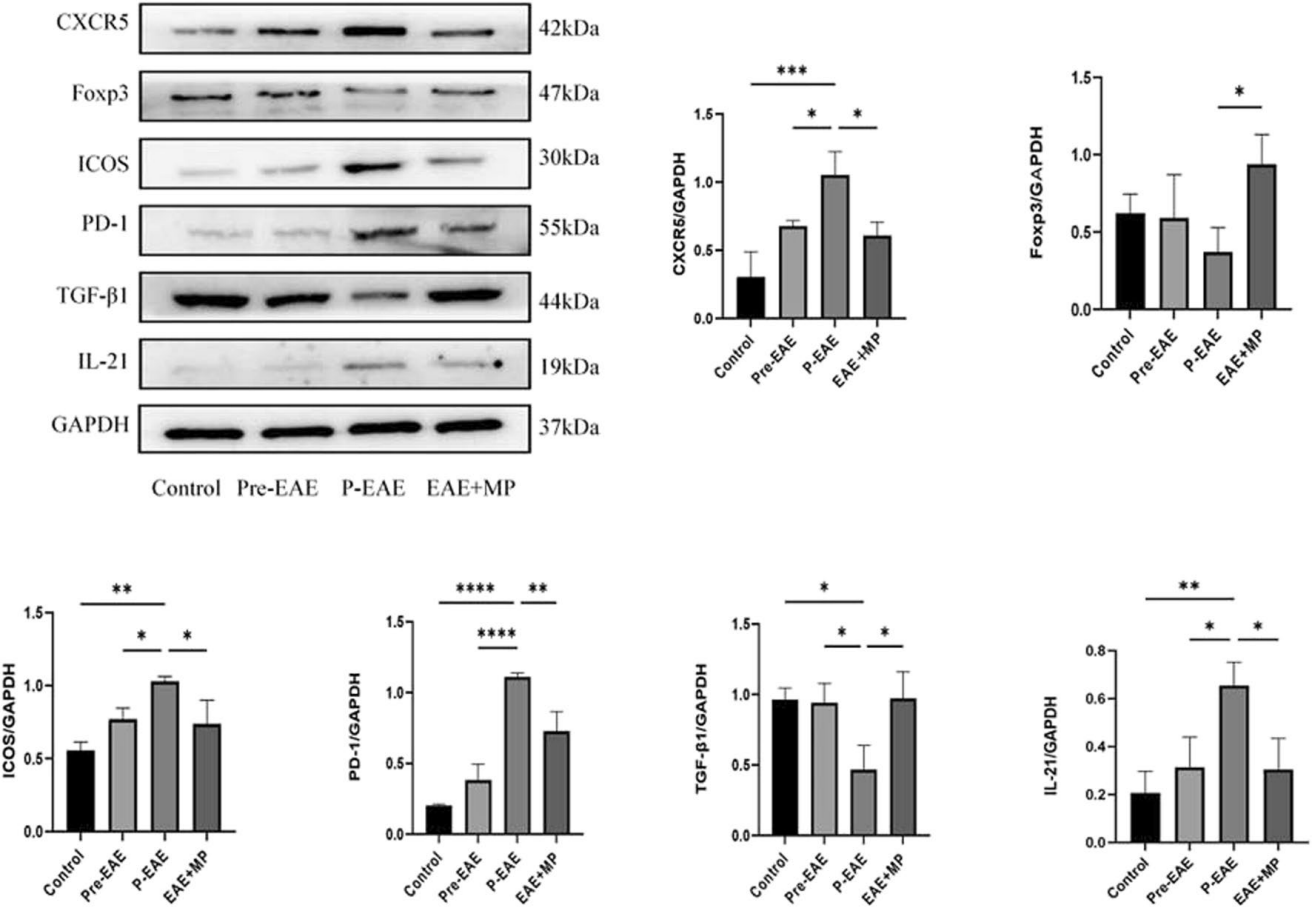
EAE mice were classified into the control group, the Pre-EAE group, the P-EAE group and the EAE + MP group. Representative blots bands of CXCR5, PD-1, iCOS, TGF-β1, FoxP3, and IL-21 in the spinal cords of EAE mice were analysed. *n* = 3 per group. The data are presented as the mean ± SEM. * *P* < 0.05, ** *P* < 0.01.*** *P* < 0.001, and **** *P* < 0.0001.

We detected various cell surface and intracellular markers associated with Tfh cells, including CXCR5, ICOS, PD-1, as well as IL-21in spinal cord tissues from the control, Pre-EAE, P-EAE, and EAE + MP groups using Western Blotting. Compared to those in the control and Pre-EAE groups, the expression levels of CXCR5, ICOS, PD-1, and IL-21 were elevated in the P-EAE group. However, the administration of MP effectively prevented the upregulation of CXCR5, ICOS, PD-1, and IL-21 protein expression observed in the P-EAE group (*P* < 0.05, Fig. 5). Furthermore, in this study, the level of TGF-β1 protein expression significantly decreased in the P-EAE group compared to that in the control and Pre-EAE groups; however, treatment with MP abrogated this reduction, especially in the P-EAE group (*P* < 0.05, Fig. 5). Moreover, the expression level of the Foxp3 protein exhibited a consistent correlation with that of TGF-β1, notably, only the EAE + MP group demonstrated a significantly elevated Foxp3 protein level compared to that of the P-EAE group (*P* < 0.05, Fig. 5).

### MP modulated Tfr/Tfh cell proliferation in EAE via the PI3K/AKT/FoxO1 and PI3K/AKT/mTOR pathways

**Fig. 6:**
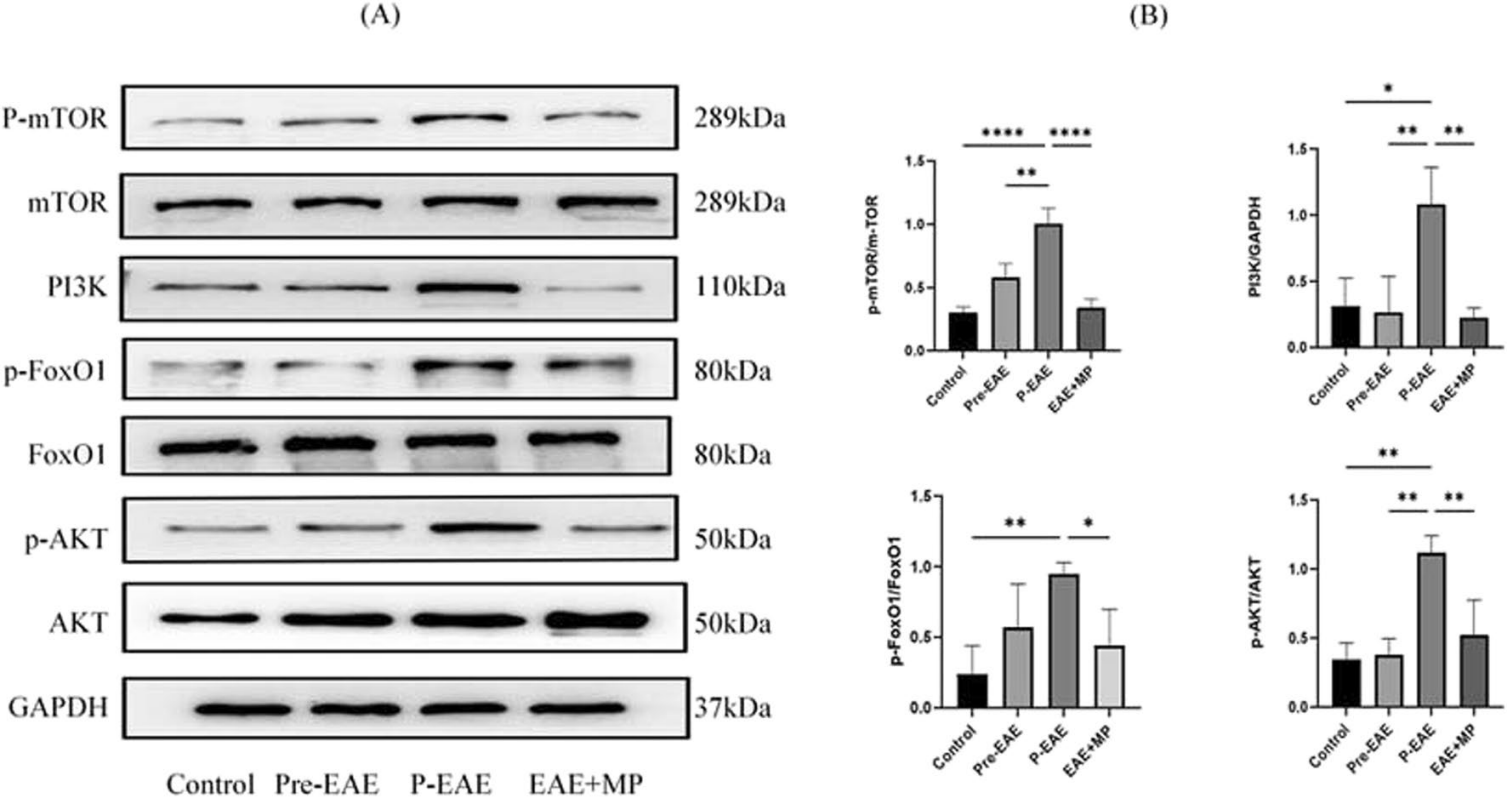
MP relieved EAE through the PI3K/AKT/mTOR and PI3K/AKT/FoxO1 signalling pathways. **A** Western blotting analysis of total PI3K, p-AKT, total AKT, p-FoxO1, total FoxO1, p-mTOR, and total mTOR. **B** Quantitative analysis revealed decreased levels of PI3K, p-AKT, p-FoxO1 and p-mTOR in the spinal cord following MP treatment. *n* = 3 per group. The data are presented as the mean ± SEM. * *P* < 0.05, ** *P* < 0.01 *** *P* < 0.001, and **** *P* < 0.0001.

To gain further insight into the molecular mechanism of MP in the treatment of EAE, we quantitatively analysed the expression of proteins associated with PI3K/AKT/FoxO1 and PI3K/AKT/ mTOR pathway. Western blot analysis revealed significantly increased greater levels of PI3K, phosphorylated AKT (p-AKT), phosphorylated FoxO1 (p-FoxO1), and phosphorylated mTOR (p-mTOR) in the P-EAE group than in the control and Pre-EAE groups. However, total AKT protein, total Foxo1 protein, and total mTOR protein did not show significantly change among the three groups. Interestingly, compared to P-EAE group, the MP treatment significantly reduced the expression of PI3K, p-AKT, p-FoxO1, and p-mTOR(*P* < 0.05, Fig. 6). This result further supports the involvement of the PI3K/AKT/FoxO1 and PI3K/AKT/mTOR pathways in P-EAE occurrence and development while highlighting that MP treatment can inhibit the activation of these signalling pathways.

## DISCUSSION

In the early phase of MS and EAE, the activation and infiltration of immune cells into the CNS, lead to disease onset and progression [38]. As MS is an autoimmune disease, immune suppressive therapies for this disease have always been the focus.

MP plays a crucial role in the current treatment of MS, and its immune regulatory properties have been gradually recognized [39]. Despite the development of numerous new drugs in the past decade, high-dose MP pulse therapy remains widely used for treating acute relapses, resulting in symptom amelioration within a few days [40]. The results showed that MP treatment ameliorated the clinical symptoms during the acute phase in EAE mice at 19 dpi (*P* < 0.05, Fig. 1). Histological analysis via HE and LFB staining revealed that MP treatment reduced inflammatory cell infiltration and demyelination in the spinal cords of EAE mice (*P* < 0.05, Fig. 1). This finding suggested that MP exerted certain anti-inflammatory and myelin-protective effects on EAE mice. During the development of MS, astrocytes and microglia can produce various cytokines and inflammatory mediators, which induce inflammation, demyelination, axonal loss and gliosis [41]. Thus, inhibiting the overactivation of microglia and astrocytes while reducing the secretion of neurotoxic cytokines represents a novel approach for MS treatment. We futher investigated the effect of MP on astrocytes and microglia in vivo. Consistent with these reports, WB analysis showed a significant increase in GFAP expression (Fig. 2A)and Iba-1 expression (Fig. 2B) in the P-EAE group, while treatment with MP significantly reduced their expression levels. These data indicate that MP can inhibit microglial activation and astrocyte reactivity in vivo. Subsequently, upregulation of the inflammatory mediator iNOS (M1 marker) and a decrease in the anti-inflammatory mediator Arg-1 (M2 marker) were observed in the spinal cords of mice with EAE compared to those in the control and Pre-EAE groups(Fig. 2C). During the progression of EAE, microglia release proinflammatory enzymes (iNOS), which may contribute to CNS damage. After MP treatment, microglia secrete anti-inflammatory enzymes (Arg-1), potentially contributing to the resolution of inflammation and tissue repair in the CNS.

To further investigate the clinical significance of our findings, we tested the effect of MP on the abundance of Tfr and Tfh cells in EAE mice. Tfr and Tfh cells are two subsets of lymphocytes with reciprocal regulatory functions involved in autoimmune diseases [42]. Tfh-like cells are involved in the pathogenesis of EAE by promoting the production of anti-MOG35-55 antibodies and forming ectopic lymphoid structures (ELSs) and GC-like structures in the CNS [35]. PD-1 serves as a key phenotypic marker for Tfh cells by regulating the long-term persistence and enhancement of memory B cells and plasma cells through survival and refinement processes at the GC while promoting IL-21 secretion [43]. ICOS is a CD28 family member costimulatory molecule expressed on T cells that upregulates CXCR5 expression on Tfh cell surfaces to promote their differentiation. Both PD-1 and ICOS expression can be used as indicators of active Tfh cell differentiation [2, 18, 26]. In this study, we observed an increase in the percentage of CD4 + CXCR5 + Foxp3-Tfh cells expressing PD-1 and ICOS during EAE progression; however, this increase abrogated following MP therapy. Tfr cells are mainly differentiated from Foxp3 + Treg precursors in the thymus and express Foxp3 and CXCR5 specifically. Tfr cells inhibit Tfh cell activity through CTLA-4 and suppress B-cell-mediated humoral immune responses in GC by inhibiting CD28 ligands [26]. Tfr cells can be distinguished from Tfh cells based on the FoxP3, CD25, and GITR expression [44, 45], and they inhibit the expansion and activity of Tfh and B cells in GCs in a CTLA-4-dependent manner [46]. We presented strong evidence showing a deficiency in Tfr cells in the spleens of untreated EAE and the reversal of this deficiency following MP therapy (Fig. 4). Our study demonstrated dysregulation of the CD4 + CXCR5 + Foxp3 + Tfr and CD4 + CXCR5 + Foxp3-Tfh cell ratios during the progression in EAE mice. We observed that MP treatment effectively restored the Tfr/Tfh ratio in the spleen of EAE mice (*P* < 0.05, Fig. 4B). Therefore, maintaining a balanced Tfr/Tfh ratio may serve as a potential therapeutic target and a valuable prognostic tool. Furthermore, there was a negative correlation between the Tfr/Tfh ratio and the IL-21 level, as well as a positive correlation with the TGF-β1 level [47]. In this study, MP treatment inhibited the expression of Tfh-related molecules (PD-1 and ICOS) and proinflammatory factors (IL-21), while increasing the expression of Tfr-related molecules (Foxp3) and anti-inflammatory factors (TGF-β1 and IL-10) in the spinal cords of EAE mice (*P* < 0.05, Fig. 5).

PI3Ks are a group of lipid kinases that exist as heterodimers and can be classified into three classes based on different stimuli [48]. AKT, a menber of the protein kinase AGC subfamily, functions as an indispensable downstream effector of PI3K [49]. The PI3K/AKT pathway has been reported to play an important role in proinflammatory factor production and release [50]. Increased activity of the PI3K/AKT pathway has been observed in autoimmune diseases such as rheumatoid arthritis [51]. FoxO1 and mTOR are downstream target proteins regulated by AKT activation. Activated AKT phosphorylates Foxo1 leading to its translocation from the nucleus to the cytoplasm, which inhibits nuclear membrane transport and transcriptional activity [52]. The PI3K/AKT/mTOR pathway drives the differentiation of CD4 + T cells through its downstream signalling cascades, where mTOR activation promotes differentiation towards Tfh cells but inhibits the generation of Tfr cells [29].

Consistent with the aforementioned reports, our study also revealed an increase in the phosphorylation of AKT, FoxO1, and mTOR in the spinal cords of EAE mice. In summary, activation of the PI3K/AKT signalling pathway and increased phosphorylation of FoxO1 and mTOR may play important roles in EAE progression. However, after MP treatment, there was a decrease in the phosphorylation level of members of this pathway (*P* < 0.05, Fig. 6).

Thus, we could presume that an imbalance in the ratio of Tfr and Tfh cells and reactivation of the PI3K/AKT pathway may contribute to the pathogenesis of EAE. Furthermore, MP not only ameliorated inflammatory cell infiltration and demyelination but also regulated the Tfr/Tfh ratio by preventing AKT, mTOR and FoxO1 from phosphorylation.

## CONCLUSION

In light of the above findings, the therapeutic efficacy exhibited of MP treatment was confirmed by a notable decrease in the extent of demyelination, inflammation, and infiltration of immune cells, as well as the activation of microglia and astrocytes. Moreover, MP increased the abundance of Tfr cells while decreasing the abundance of Tfh cells in EAE mice, as well as restored the balance between Tfr/Tfh-type cytokines (TGF-β1 and IL-21). Mechanistically, MPs may regulate Tfr and Tfh cells by inhibiting the PI3K/ AKT/FoxO1 and PI3K/AKT/mTOR signalling pathways.

## Supplementary Information

Below is the link to the electronic supplementary material.

Supplementary file1 Supplementary Figure 1: Isotype control of ICOS, PD-1, and Foxp3. (JPG 300 KB)

Supplementary file2 Supplementary Figure 2: Simple staining of ICOS, PD-1, and Foxp3. (JPG 363 KB)

## Data Availability

The datasets generated or analyzed during this study are available from the corresponding author on reasonable request.
