# Supplementary figures and images for: Methylprednisolone Modulates the Tfr/Tfh ratio in EAE-Induced Neuroinflammation through the PI3K/AKT/FoxO1 and PI3K/AKT/mTOR Signalling Pathways
Source: Inflammation. 2024 Jul 9;48(2):950–62. doi: 10.1007/s10753-024-02099-y (PMC12052871; doi:10.1007/s10753-024-02099-y)

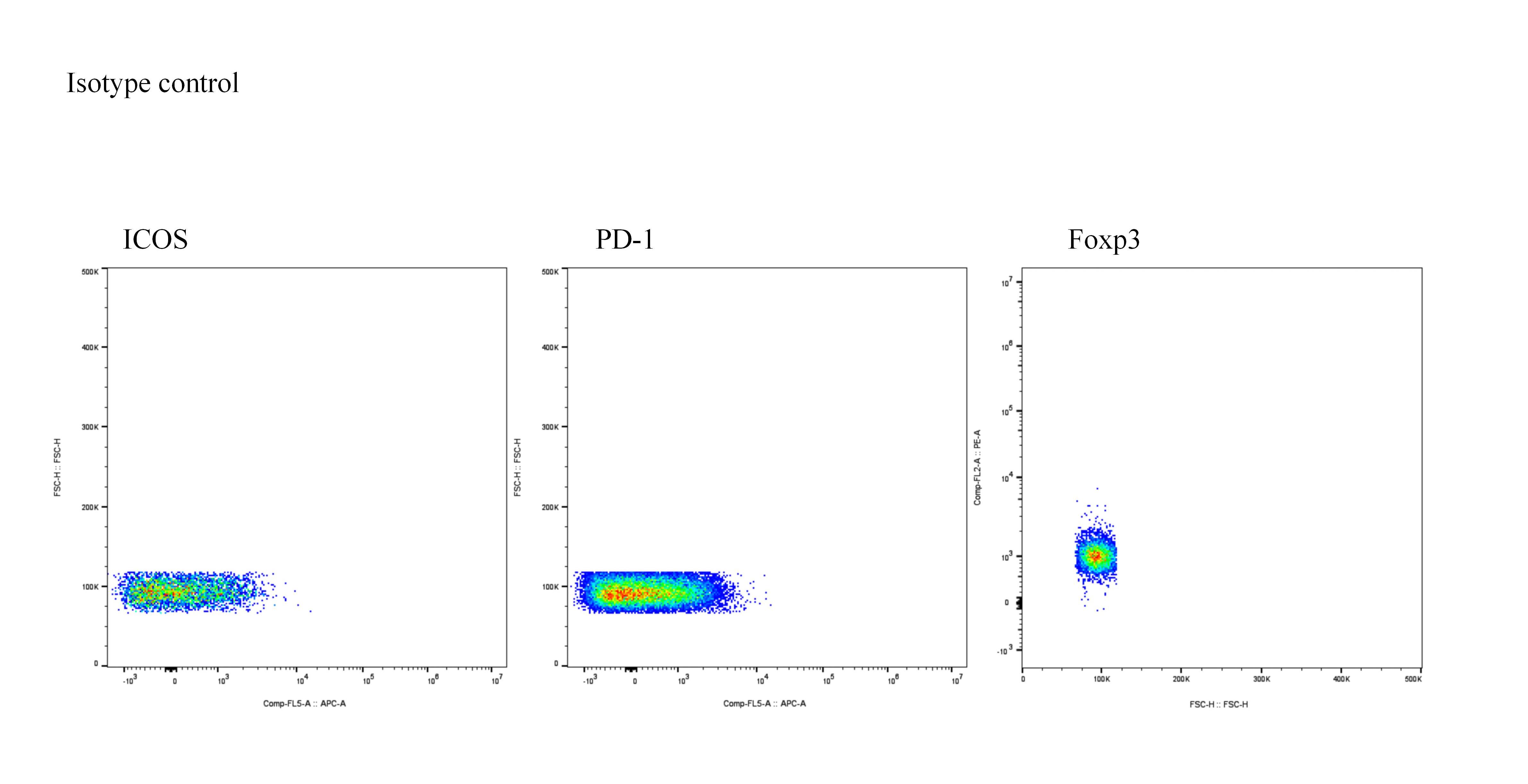

Supplement: Supplementary file 1 — Supplementary file1 Supplementary Figure 1: Isotype control of ICOS, PD-1, and Foxp3. (JPG 300 KB) [file 10753_2024_2099_MOESM1_ESM.jpg]

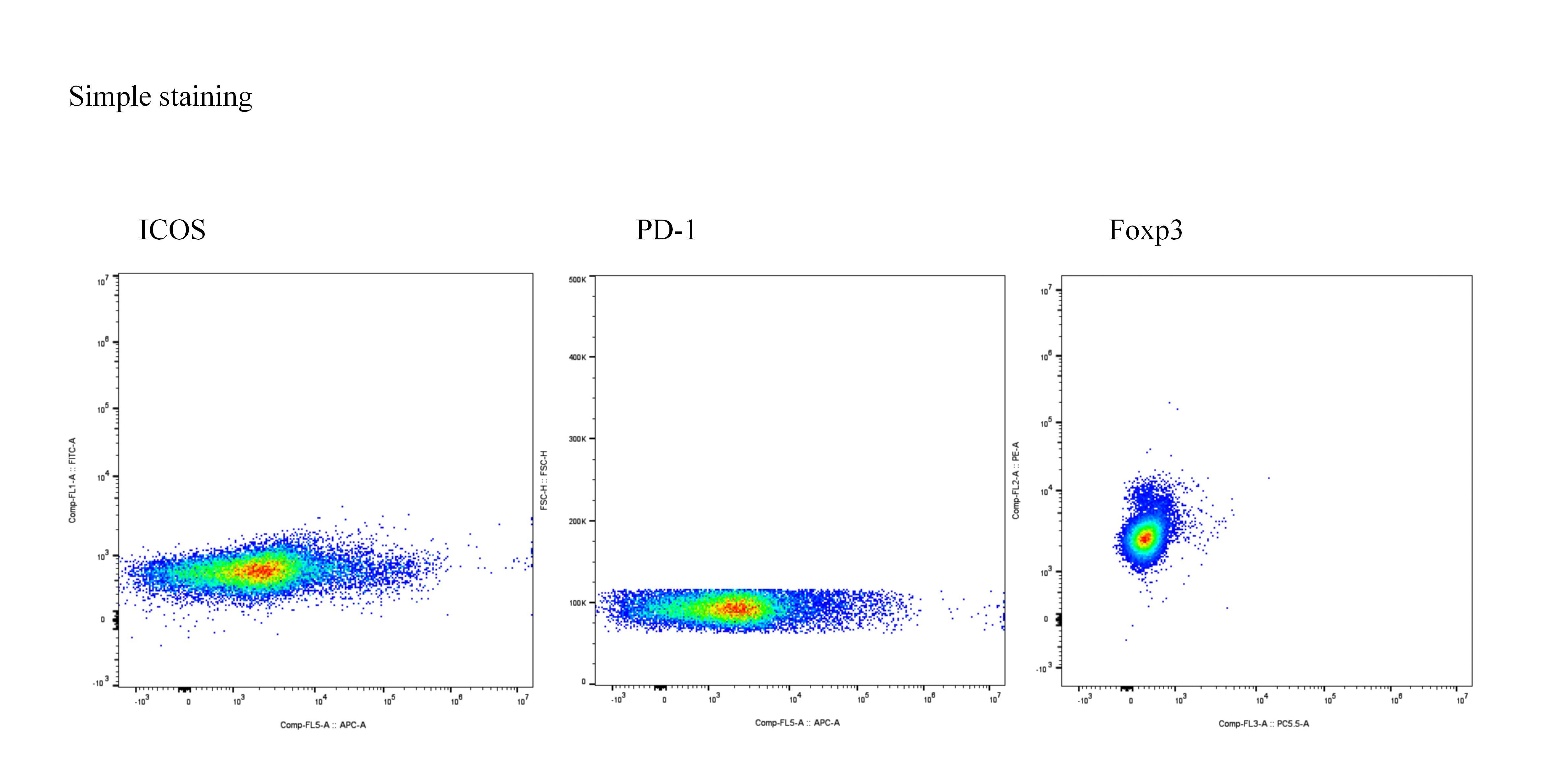

Supplement: Supplementary file 2 — Supplementary file2 Supplementary Figure 2: Simple staining of ICOS, PD-1, and Foxp3. (JPG 363 KB) [file 10753_2024_2099_MOESM2_ESM.jpg]
